# Mr. Lunn, on Removing a Scirrhous Parotid Gland

**Published:** 1809-09

**Authors:** William Lunn

**Affiliations:** Clayfield Hill, near Rotheram


### *To the Editors of the Medical and Phyſical Journal.*

Gentlemen,

I Conceive nothing adds more to the advancement of medical or chirurgical knowledge than the plain statement of rare cases, with their treatment, whether attended by success or failure; and as the object of your valuable miscellany is to diffuse widely the various improvements of our profession, fession, I hope the following case of Scirrhous Parotid Gland, successfully extirpated, will be thought worthy of insertion.

In sending this forth to the public, I do not pretend to any new operation, for we find it mentioned as far back as Heister, who says, he has "happily removed many parotid and submaxillary glands;" but his directions for the operation and cure, appear to me more calculated to deter, than encourage the operator; indeed, from our increased knowledge of anatomy and physiology, no surgeon of the present day can possibly profit by the rules he has laid down.*

Whether the parotid gland has been removed by any of our modern surgeons I know not, I never saw it done, nor have I met with any of my medical friends who have, yet I can assure those who may meet with a case similar to mine, that it may be done very safely and effectually, by a steady careful dissection, and a clear anatomical knowledge of the parts.

In February last a cottager's wife, the mother of three children, and again pregnant, aged 26, of healthy stamina, consulted me for a large oblong prominent tumour extending from under the lobe of the right ear, to about an inch below the angle of the lower jaw, of which she gave the following account. In November, 1803, she discovered a small tumour under the ear, when recovering from a tedious confinement of her first child; but as it was without pain, she took no notice of it for two years, when the increase in size rather than any uneasiness, caused her to apply for medical aid; various topical applications were used without effect; what they were I am not acquainted.

On examination I found an irregular knotty tumour, with a prickling lancinating pain, and enlarged veins meandering on its surface. From its situation and strong attachment at the upper end, there couldnot be a doubt of its being the parotid gland; and whether to remove it, or leave my patient to languish to that ultimatum of misery, the universal effect of cancer, I at first wavered. Having made up my mind, March 9th, I removed the disease by making an incision through the integuments, the whole length of the tumour, a little circular; I then made a second at the same point as the first, giving it an opposite direction, and united with the other in the inferior part, figuring an oval, careful to preserve sufficient skin to cover the wound when the

* Vide, Heister's Surgery, p. 46, Part II.

the tumour should be removed; I then dissected away the integuments, and as I found the least attachment at its lower end, I raised the tumour with a small hook and dissected it upwards, taking up the vessels as I divided them; in this I found no difficulty, except in two arteries which lay deep between the condyloid process of the lower jaw, and the mastoid process of the temporal bone; these I conceived to be the temporalis externa, and the tranversalis faciei. This part of the operation was rather perplexing, from the deep and very firm attachment of the tumour under the ear, yet without any considerable loss of blood, I succeeded in securing the bleeding vessels with a small curved needle; I then brought the lips of the wound together by sutures and adhesive plaster, applying a soft thick pledgit of tow, spread with the cerat. lapid. calam. and a calico bandage over the whole.

I will not take up your time with a detail of each day's appearances during the cure, suffice to say it healed kindly without any thing particular occurring, except a small abscess, which I opened; the ligatures, seven in number, came away in due time, and the wound was completely healed on the 20th day after the operation.

The effects of the operation were such as might be expected on the division of the pes anserinus; that side became immediately paralytic, with the mouth drawn to the opposite side of the face*; my patient complained of great numbness, and often a painful sensation during the cure, as if nettled violently.

August 1st, These unpleasant symptoms are entirely gone off; still she has an imperfect use of the muscles both in mastication and articulation, though very evidently improved; the mouth has resumed its proper place, and the eyelids have regained their sensation and action completely; she says that side of her face is generally colder than the other; she does not experience any want of saliva in chewing her food; the muscles connected with the operation do not appear to have lost any of their plumpness, and except when she speaks, no difference can be observed in the two sides of the face.

I am, &c.

WILLIAM LUNN.

*Clayfield Hill, near Rotheram,*

*August* 10, 1809.

---

* It appears strange if Heister ever removed the Parotid Gland, that he did not mention this circumstance, which must unavoidably happen in this operation.

